# Variations of Prevalence and Incidence of Atrial Fibrillation and Oral Anticoagulation Rate According to Different Analysis Approaches

**DOI:** 10.1038/s41598-018-25111-6

**Published:** 2018-05-01

**Authors:** Pil-Sung Yang, Soorack Ryu, Daehoon Kim, Eunsun Jang, Hee Tae Yu, Tae-Hoon Kim, Jinseub Hwang, Boyoung Joung, Gregory Y. H. Lip

**Affiliations:** 1 Department of Cardiology, CHA Bundang Medical Center, CHA University, Seongnam, Korea; 2 Department of Computer Science and Statistics, Daegu University, Daegu, Korea; 3 Division of Cardiology, Department of Internal Medicine, Yonsei University Health System, Seoul, Korea; 4 Institute of Cardiovascular Sciences, University of Birmingham, Birmingham, United Kingdom

## Abstract

The reported incidence and prevalence of atrial fibrillation (AF) has been inconsistent across published studies. Using the National Health Insurance Service database of Korea, the prevalence and incidence of AF, and oral anticoagulation (OAC) use of AF patients were explored according to three different approaches; ‘formal approach’, considering individual AF diagnosis and mortality; ‘limited diagnosis approach’, using upper 5 main diagnosis; and ‘medical use approach’, using the number of medical use AF population by year without considering individual AF history and mortality. The AF prevalence progressively increased by 2.46-fold from 0.50% in 2004 to 1.54% in 2015 when using a ‘formal approach’ (p for trend <0.001). The overall prevalence was 1.09% and 0.97% when using a ‘formal approach’ and ‘limited diagnosis approaches’, respectively. Overall prevalence decreased to 0.52% with a ‘medical use approach’. The trend of annual AF incidence was stable when using a ‘formal approach’, but increased by 15% when using a ‘medical use approach’. OAC rate in 2015 was 2.1 times higher when using a ‘medical use approach’ compared to using a ‘formal approach’ (40.3% vs. 19.1%, p < 0.001). Given the wide variability in prevalence and incidence figures with different analysis approaches, careful attention to the analysis methodology is needed.

## Introduction

Atrial fibrillation (AF) is the most common sustained cardiac arrhythmia in the general population^1^. Due to ageing of the general population and the increasing prevalence of risk factors, there has been an increase in the incidence and prevalence of AF^2^. However, estimates of AF incidence and prevalence differ widely. These variations might be attributable to the design and time period of study, and an improved surveillance for AF with increased use of diagnostic tools and health care awareness. This study investigated the prevalence and incidence of AF population according to different analysis methodological approaches using national insurance data. Second, we examined the variation of national oral anticoagulation (OAC) rate of AF population according to the variation of prevalence of AF.

## Methods

### Study population

This nationwide study is based on the national health claims database established by the National Health Insurance Service (NHIS) of Korea^3^. The NHIS is the single insurer managed by the Korean government, and the majority (97.1%) of Korean population are mandatory subscribers, with the remaining 3% of the population being medical aid subjects. The NHIS database contains the information of medical aid subjects, therefore it is based on the entire Korean population. The following medical information is provided: patients’ sociodemographic information, their use of inpatient and outpatient services, pharmacy dispensing claims, and mortality data. This study was approved by the Institutional Review Board of Yonsei University Health System (4-2016-0179). The IRB waived the requirement to obtain informed consent, and this study was conducted in accordance with the tenets of the Declaration of Helsinki.

### Definition of atrial fibrillation

AF was diagnosed using the International Classification of Disease 10th Revision (ICD-10) codes, I48 (atrial fibrillation and atrial flutter), I48.0 (atrial fibrillation), and I48.1 (atrial flutter). We regarded first date of getting AF-related ICD-10 codes as the newly diagnosed year, and excluded first 2 years (2002 to 2003) to avoid the possibility for misdiagnosis of preexisting AF for incident AF. Moreover, patients were defined as AF only when it was a discharge diagnosis or confirmed at least twice in the outpatient department to ensure diagnostic accuracy. The AF diagnosis has previously been validated with a positive predictive value of 94.1%^4,5^. We excluded patients <20 years, patients with valvular AF. Valvular AF was defined from a diagnosis of mitral stenosis (ICD-10: I05.0, I05.2, and I34.2) or prosthetic heart valve (ICD-10: Z95.2–Z95.4), and insurance claims for valve replacement or valvuloplasty.

### Definition of comorbidities

Comorbidities were defined using the medical claims according to ICD-10 codes and prescription medication use. In order to ensure diagnostic accuracy, we defined patients with comorbidities (including previous stroke, previous transient ischaemic attack, heart failure, hypertension, diabetes, previous myocardial infarction, peripheral arterial disease, dyslipidemia) when it was a discharge diagnosis or was confirmed at least twice in an outpatient setting, which was similar to previous studies with NHIS^4–9^. The definitions of comorbidities are presented in Supplementary Table S1.

### Methodological approaches

We evaluated three different methodological approaches to evaluate the prevalence and incidence of AF; ‘formal approach’, ‘limited diagnosis approach’, and ‘medical use approach’. (1) In the ‘formal approach’, we considered individual AF diagnosis history and mortality. The annual prevalence of AF was calculated by dividing the number of AF patients of each year with exception for AF patients who died in previous year by the number of total Korean residents of that year. The annual incidence of AF was the number of incident cases of AF divided by the number of person-years at risk among all Korean residents of that year who had never been diagnosed as AF. Supplementary Table S2 shows the number and distribution of total Korean residents aged ≥20 years. (2) In the ‘limited diagnosis approach’, we included patients with an AF diagnosis within 5 main diagnosis fields during whole follow up period. The other conditions were the same as in the ‘formal approach’. (3) In the ‘medical use approach’, we used the number of AF patients who claimed medical expenses by year without considering individual AF history and mortality. We calculated the annual prevalence of AF by the number of AF patients who claimed medical expenses in each year divided by the number of total Korean residents of that year. The incident AF of each year was calculated using an increase in the number of AF patients who claimed medical expenses as compared to the previous year. The annual incidence of AF was the number of incident AF divided by the number of Korean residents of that year. The OAC rate was also calculated based on prevalence by the three different approaches.

### Statistical analysis

Data are presented as mean value ± standard deviation for continuous variables and proportions for categorical variables. The Cochran-Armitage trend test was used for analyzing temporal trends of categorical variables. The nonparametric test for trend by Jonckheere-Terpstra was used for continuous variables. All tests were two-tailed, with P < 0.05 considered significant. Statistical analyses were conducted with SAS version 9.4 (SAS Institute, Cary, NC, USA), R version 3.4.1, and SPSS version 23.0 statistical package (SPSS Inc., Chicago, IL, USA).

### Data availability

The data that support the findings of this study are available from the NHIS, but restrictions apply to the availability of these data, which were used under license for the current study, and so are not publicly available. Data are however available from the authors upon reasonable request and with permission of the NHIS.

## Results

Between January 1, 2004 and December 31, 2015, the ‘formal approach’ identified 802,503 patients with newly diagnosed AF. The mean age was 64.9 ± 14.9 years, and 52.9% were men. Table 1 shows temporal trends in demographics and comorbidities of patient with AF.

**Table 1:** Baseline characteristics of incident AF between 2004 and 2015.

	Overall 2004–2015 (n = 802,503)	2004–2007 (n = 258,638)	2008–2011 (n = 265,386)	2012–2015 (n = 278,479)	p-value
Age (years)	64.9 ± 14.9	63.0 ± 14.9	64.8 ± 14.9	66.9 ± 14.6	<0.001
Male	52.9	52.1	52.6	53.9	<0.001
Comorbidity					
Previous stroke	16.9	11.7	17.4	21.2	<0.001
Previous TIA	6.7	3.8	7.2	9.0	<0.001
Heart failure	21.3	18.2	21.0	24.3	<0.001
Hypertension	65.5	57.1	66.8	71.9	<0.001
Diabetes mellitus	20.6	17.1	20.9	23.7	<0.001
Previous MI	7.3	6.1	7.5	8.3	<0.001
PAD	9.4	4.3	9.5	13.9	<0.001
Dyslipidemia	47.4	29.0	47.8	64.0	<0.001
CHA_2_DS_2_-VASc score	2.96 ± 2.10	2.53 ± 1.87	2.99 ± 2.09	3.34 ± 2.22	<0.001

AF; atrial fibrillation; TIA, transient ischaemic attack; MI, myocardial infarction; PAD, peripheral artery disease.

Values are presented as % or mean ± SD.

**Figure 1:**
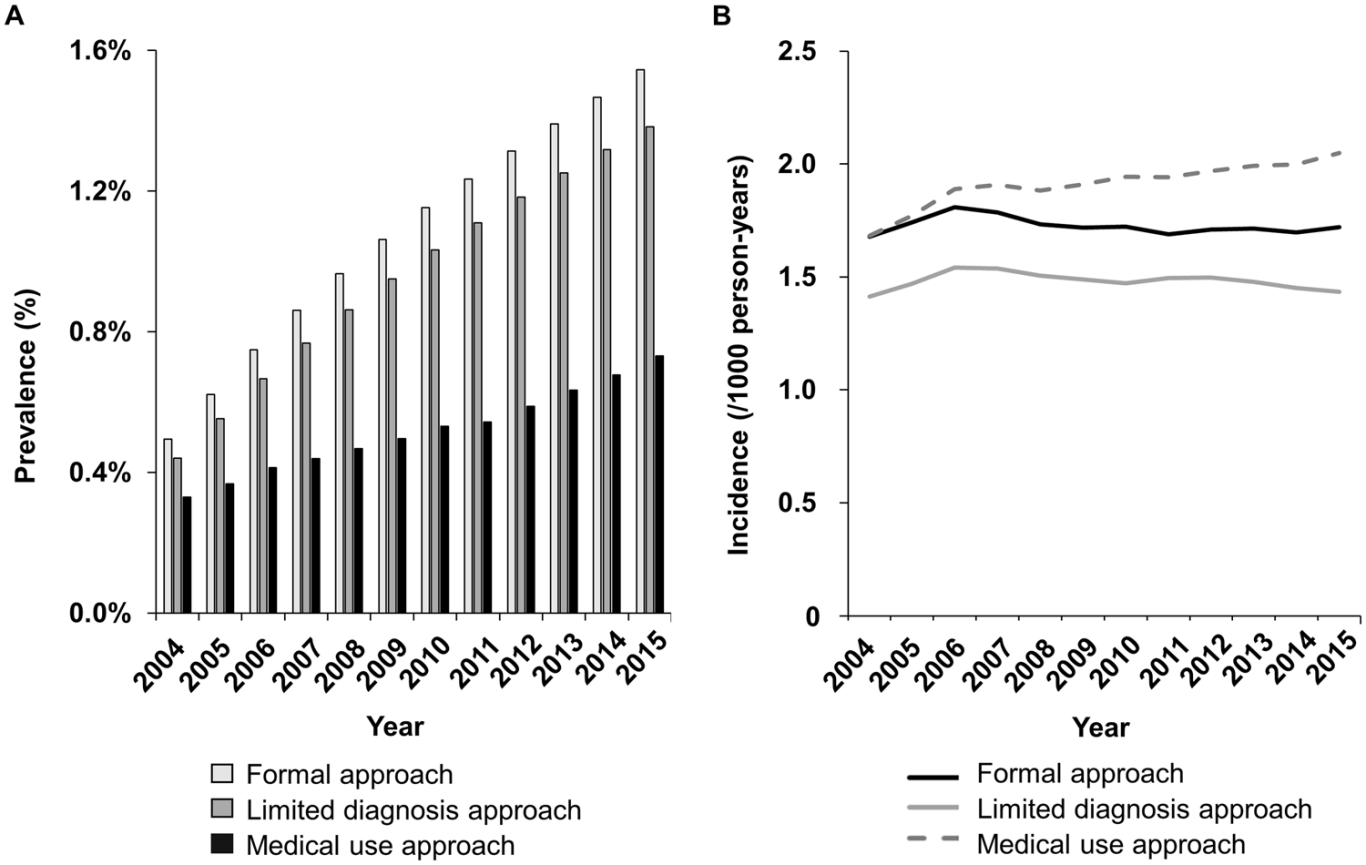
Annual prevalence (**A**) and incidence (**B**) of atrial fibrillation between 2004 and 2015 according to different analysis approaches.

Figure 1A shows the annual prevalence of AF between 2004 and 2015. The prevalence progressively increased by 2.46-fold from 0.50% in 2004 to 1.54% in 2015 when using a ‘formal approach’ (p for trend <0.001). The overall prevalence of AF was 1.09% and 0.97% when using a ‘formal approach’ and a ‘limited diagnosis approach’, respectively. When using a ‘medical use approach’, the overall prevalence was decreased to 0.52%, although the annual prevalence still gradually increased (p for trend <0.001). Figure 1B shows the annual incidence of AF between 2004 and 2015. During the study period of 12-years, the incidence did not change significantly with the incidence of 1.68 in 2004 and 1.73 per 1,000 person-years in 2015 when using a ‘formal approach’. The incidence dramatically increased from 1.7 in 2004 to 2.0 per 1,000 person-years in 2015 when using a ‘medical use approach’ (p for trend <0.001).

**Figure 2:**
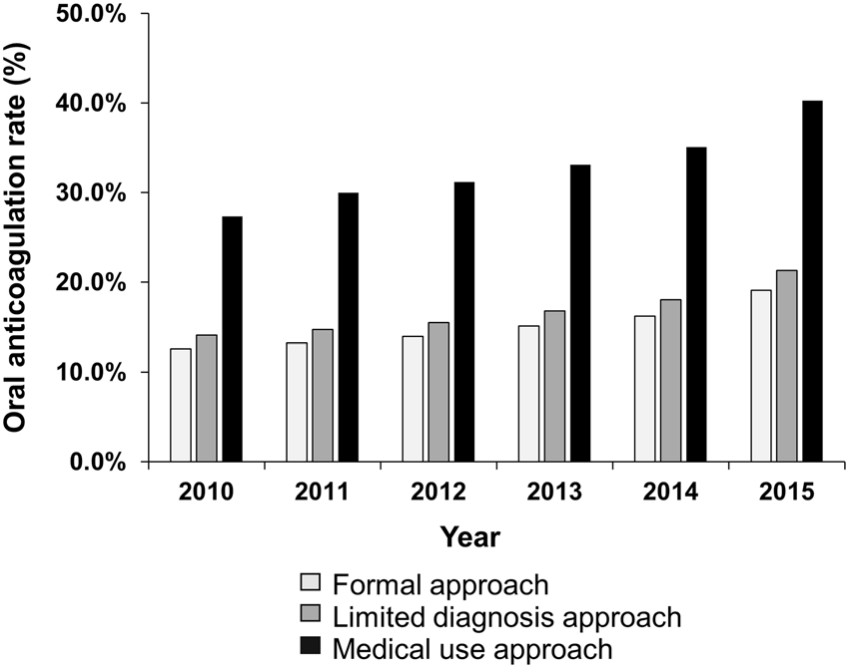
The change of oral anticoagulation rate according to different analysis approaches in overall atrial fibrillation population.

Figure 2 shows OAC rates according to different analysis approaches in overall AF population. The rates of OAC use at the last year (in 2015) were 19.1%, 21.3% and 40.3% in overall AF population when using ‘formal approach’, ‘limited diagnosis approach’, and ‘medical use approach’, respectively. The rate of OAC use in 2015 was 2.1 times higher when using the ‘medical use approach’ compared to using the ‘formal approach’ (p < 0.001).

## Discussion

In this study, the prevalence and incidence of AF changed dramatically by three different analysis approaches. The ‘formal approach’ showed a progressive increase of prevalence and stable incidence. The ‘medical use approach’ showed progressive increase of both prevalence and incidence. The overall prevalence of ‘medical use approach’ was only half of that with the ‘formal approach’. The ‘formal approach’ was used in most of the recent studies about the AF epidemiology, including nationwide cohort based studies from Taiwan and Korea^8,10^. On the other hand, Lee *et al*. used the ‘medical use approach’ to investigate the incidence and prevalence of AF in Korea^11,12^.

The prevalence of AF calculated using ‘formal approach’ in our study was similar to the recent prevalence rates ranging from 1.07% to 1.6% in Asia (1.07% in Taiwan^10^, 1.38% in Korea^8^, 1.5% in Singapore^13^, and 1.6% in Japan^14^). Therefore, the prevalence using ‘medical use approach’, which is half of that with the ‘formal approach’, is likely to be underestimated, and not to reflect the actual prevalence.

The overall AF incidence of Korean was around 1.70 per 1,000 person-years using the ‘formal approach’ which was similar with the recently reported incidence of Taiwan of 1.51 per 1,000 person-years^10^, and was lower compared to that seen in Caucasians^2,15,16^. Using the ‘formal’ and ‘limited diagnosis approach’, the annual trends of AF incidence were stable similarly with the recent Taiwan study, but were clearly increased when using the ‘medical use approach’^10^.

Using national insurance data, which includes information on the medical use of entire citizens, it is possible to study various national medical indicators. However, depending on how the data is analysed, the basic epidemiology indicators such as prevalence and incidence may also vary as we have shown in this study. If prevalence is miscalculated, other important medical indicators will be misjudged accordingly. In our results, the national rate of OAC use in AF patients differed by more than two-fold depending on the method we used to calculate the prevalence. A careful approach is needed when conducting this kind of research.

### Study limitations

There are several limitations in this study, given the natures of the nationwide registry dataset we used. Although administrative databases are increasingly used for clinical research, such studies are potentially susceptible to errors arising from coding inaccuracies. To minimize this problem, we examined the nationwide cohort, and applied the definition that we already validated in previous studies that used a Korean NHIS sample cohort^4,5,9,17^. Since we defined AF cases only with ICD-10 codes, it is possible that either paroxysmal or asymptomatic AF cases, which were not ascertained by these codes, were not recorded.

## Conclusions

The reported incidence and prevalence of AF varies widely when using different analysis approaches. Compared with the ‘formal approach’, the ‘medical use approach’ showed an increasing trend of incidence, half the prevalence of AF, and twofold higher OAC use. Given the wide variability in prevalence and incidence figures with different analysis approaches, careful attention to the analysis methodology is needed.

## Electronic supplementary material

Supplementary Tables
