# Supplementary material for: Variations of Prevalence and Incidence of Atrial Fibrillation and Oral Anticoagulation Rate According to Different Analysis Approaches
Source: Sci Rep. 2018 May 1;8:6856. doi: 10.1038/s41598-018-25111-6 (PMC5931533; doi:10.1038/s41598-018-25111-6)
Supplement: Supplementary file 1 — Supplementary Tables [file 41598_2018_25111_MOESM1_ESM.doc]

**Supplementary Information**

**Variations of Prevalence and Incidence of Atrial Fibrillation and Oral Anticoagulation Rate According to Different Analysis Approaches**

Pil-Sung Yang, MD*;^1^ Soorack Ryu*;^2^ Daehoon Kim, MD*;^3^ Eunsun Jang;^3^ Hee Tae Yu, MD;^3^ Tae-Hoon Kim, MD;^3^ Jinseub Hwang†;^2^ Boyoung Joung, MD†;^3^ Gregory Y.H. Lip, MD†^4^

From Department of Cardiology, CHA Bundang Medical Center, CHA University, Seongnam, Korea,^1^ Department of Computer Science and Statistics, Daegu University, Daegu, Korea,^2^ Division of Cardiology, Department of Internal Medicine, Yonsei University Health System, Seoul, Korea,^3^ and Institute of Cardiovascular Sciences, University of Birmingham, United Kingdom.^4^

[*These three authors contributed equally to this work]

[†Joint senior authors]

**Supplementary Table S1. ICD-10 codes used for defining the comorbidities.**

| **Comorbidities** | **Definitions** | **Codes or conditions** |
| --- | --- | --- |
| Previous stroke | Defined from diagnosis* | ICD-10: I63, I64 |
| Previous TIA | Defined from diagnosis* | ICD-10: G45 |
| Heart failure | Defined from diagnosis* | ICD-10: I11.0, I50, I97.1 |
| Hypertension | Defined from diagnosis* | ICD-10: I10, I11, I12, I13, I15 |
| Diabetes mellitus | Defined from diagnosis* plus treatment | ICD-10: E10, E11, E12, E13, E14 plus treatment: all kinds of oral anti-diabetics and insulin |
| Previous MI | Defined from diagnosis* | ICD-10: I21, I22, I25.2 |
| Peripheral artery disease | Defined from diagnosis* | ICD-10: I70.0, I70.1, I70.2, I70.8, I70.9 |
| Dyslipidemia | Defined from diagnosis* | ICD-10: E78 |
| Valvular atrial fibrillation | Defined from any diagnoses of mitral stenosis or heart valve surgery | ICD-10: I05.0, I05.2, I34.2, Z95.2-4 or insurance claims for valve replacement or valvuloplasty |

MI, myocardial infarction; TIA, transient ischaemic attack.

*To ensure accuracy, comorbidities were established based on one inpatient or two outpatient records of ICD-10 codes in the database.

**Supplementary Table S2. Distribution of Korean National Health Insurance beneficiaries aged ≥ 20 years by age and sex.**

|  | **2004** | **2005** | **2006** | **2007** | **2008** | **2009** | **2010** | **2011** | **2012** | **2013** | **2014** | **2015** |
| --- | --- | --- | --- | --- | --- | --- | --- | --- | --- | --- | --- | --- |
| Total, n | 36,316,172 | 36,778,840 | 37,053,694 | 37,594,207 | 38,078,786 | 38,547,087 | 39,007,928 | 39,557,910 | 40,056,827 | 40,593,174 | 41,166,634 | 41,701,269 |
| Sex group |  |  |  |  |  |  |  |  |  |  |  |  |
| Men, n | 17,966,021 | 18,201,322 | 18,330,126 | 18,599,199 | 18,846,486 | 19,084,141 | 19,322,618 | 19,603,998 | 19,849,037 | 20,129,689 | 20,435,976 | 20,711,331 |
| Women, n | 18,350,151 | 18,577,518 | 18,723,568 | 18,995,008 | 19,232,300 | 19,462,946 | 19,685,310 | 19,953,912 | 20,207,790 | 20,463,485 | 20,730,658 | 20,989,938 |
| Age group, n |  |  |  |  |  |  |  |  |  |  |  |  |
| 20-29 | 7,844,509 | 7,717,886 | 7,611,094 | 7,461,124 | 7,378,678 | 7,189,265 | 7,003,238 | 6,883,527 | 6,792,802 | 6,766,617 | 6,835,393 | 6,896,972 |
| 30-39 | 8,993,631 | 8,920,273 | 8,857,665 | 8,800,441 | 8,608,104 | 8,522,592 | 8,449,178 | 8,358,487 | 8,275,072 | 8,147,676 | 7,976,119 | 7,857,933 |
| 40-49 | 8,342,269 | 8,440,435 | 8,932,912 | 8,932,912 | 8,932,912 | 8,932,912 | 8,932,912 | 8,932,912 | 8,932,912 | 8,932,912 | 8,932,912 | 8,932,912 |
| 50-59 | 4,942,069 | 5,318,553 | 5,573,511 | 5,889,864 | 6,198,789 | 6,575,316 | 7,028,311 | 7,508,879 | 7,788,986 | 8,041,766 | 8,263,032 | 8,392,795 |
| 60-69 | 3,705,736 | 3,724,224 | 3,733,515 | 3,872,908 | 3,987,188 | 4,072,221 | 4,166,825 | 4,197,449 | 4,314,938 | 4,466,479 | 4,712,416 | 5,089,893 |
| 70-79 | 1,839,419 | 1,973,338 | 2,072,709 | 2,208,766 | 2,338,886 | 2,484,188 | 2,603,758 | 2,755,942 | 2,954,146 | 3,056,411 | 3,129,667 | 3,159,341 |
| ≥ 80 | 648,539 | 684,131 | 707,996 | 758,882 | 815,194 | 879,662 | 941,677 | 1,002,315 | 1,078,184 | 1,159,595 | 1,259,996 | 1,371,423 |
